# A Review of Approaches to Detecting Malingering in Forensic Contexts and Promising Cognitive Load-Inducing Lie Detection Techniques

**DOI:** 10.3389/fpsyt.2018.00700

**Published:** 2018-12-21

**Authors:** Jeffrey J. Walczyk, Nate Sewell, Meghan B. DiBenedetto

**Affiliations:** Psychology and Behavioral Sciences, Louisiana Tech University, Ruston, LA, United States

**Keywords:** malingering detection techniques, cognitive malingering detection, theory of mind, forensic psychiatry, inducing cognitive load

## Abstract

Malingering, the feigning of psychological or physical ailment for gain, imposes high costs on society, especially on the criminal-justice system. In this article, we review some of the costs of malingering in forensic contexts. Then the most common methods of malingering detection are reviewed, including those for feigned psychiatric and cognitive impairments. The shortcomings of each are considered. The article continues with a discussion of commonly used means for detecting deception. Although not traditionally used to uncover malingering, new, innovative methods are emphasized that attempt to induce greater cognitive load on liars than truth tellers, some informed by theoretical accounts of deception. As a type of deception, we argue that such cognitive approaches and theoretical understanding can be adapted to the detection of malingering to supplement existing methods.

The present article is partly a review of methods of detecting malingering. Previous reviews of malingering detection methods include Sartori et al. (1) as well as Sartori et al. (2). The present review adds uniquely to the literature by highlighting recent cognitive-based methods of lie detection and relevant theory potentially applicable to malingering detection.

The Diagnostic and Statistical Manual of Mental Disorders, Fifth Edition (DSM-V) defines malingering as “the intentional production of false or grossly exaggerated physical or psychological symptoms, motivated by external incentives” [(3), p. 726]. Although the concept of malingering has existed for centuries, it was not until the mid-1900's that the term “malingering” was introduced to refer to soldiers who feigned illness or disability in order to avoid military service (4). The term's usage has broadened to include other incentives, such as avoiding work, gaining financial advantage, avoiding arrest, evading criminal prosecution, mitigating sentencing, receiving medication, or gaining admission to a hospital for shelter (3, 5). Despite a clear definition, the detection of malingering is elusive. For instance, Rogers and Shuman (6) found that the use of DSM criteria results in the accurate identification of only 13.6–20.1% of actual malingerers (true positives). However, 79.9–86.4% of individuals are misclassified as malingerers (false positives) using the same criteria. The accurate detection of malingering is thus a pressing societal issue.

## Negative Effects of Misclassification/Burden on the Criminal-Justice System

In addition to the problem of not identifying individuals who are malingering, there are also very serious consequences for misclassifying malingering when an individual's presentation is genuine (false positives). Labeling an individual as a malingerer can be stigmatizing, which carries negative connotations and can negatively impact individuals for the remainder of their lives (7). In addition, many clinicians avoid diagnosing malingering for fear of legal consequences. Because of the difficulty of arriving at an accurate diagnosis, these clinicians fear they will be sued and are, therefore, reluctant to apply the label (8).

In the criminal-legal realm, malingering has a negative impact on the proper execution of justice. Failure to detect malingering in cases of insanity or incompetency can delay prosecution for months or years and often results in unnecessary hospitalizations. It also provides malingerers with the opportunity to be moved from secure facilities, such as jails or prisons, to psychiatric facilities with more comfortable environments and from which escape is easier (9). Prison inmates also feign psychiatric or cognitive symptoms in order to transfer to medical centers where they can gain access to pain medication and have greater contact with female staff (10, 11). Some researchers have expressed concern regarding the inappropriate use of antipsychotic medications administered to inmates who successfully feign psychosis. In addition to being very costly, such medications can cause harmful side-effects such as dystonias, diabetes, high blood cholesterol, and metabolic syndrome (12).

In summary, despite ongoing advances in malingering detection, many individuals successfully malinger mental, cognitive, and physical disorders in order to gain financial compensation, avoid work, gain access to medications, and avoid prison. This places a large financial burden on society, negatively impacts the efficient operation of the healthcare system, and increases medical costs. The creation or discovery of new and effective malingering detection methods has the potential to significantly reduce the burden of malingering on the criminal justice system and on society generally.

## Current Assessments of Malingering

### Measuring Malingering Detection Accuracy

The detection of malingering is typically done using standardized assessments as this approach gives clinicians access to the most current and scientifically-based methods (13). Malingering detection accuracy is assessed by evaluating each measure's *sensitivity, hit rate, positive predictive power* (PPP), *and negative predictive power* (NPP). Sensitivity refers to the ability of a measure to accurately identify individuals who have the condition the measure is designed to detect. Specificity is the ability of a measure to identify individuals for whom the condition is not present. The hit rate is the total proportion of accurately identified cases, i.e., the true positives plus the true negatives (14–16). PPP is the percentage of individuals detected as malingering who are actually malingering, while the NPP is the percentage of honest individuals (17, 18).

### Psychiatric and Cognitive Malingering Detection Strategies

Rogers et al. validated 10 strategies for the detection of feigning within the domain of mental disorders (19, 20). These strategies fall into two broad categories: *unlikely presentations* and *amplified presentations* (21). Table 1 provides a description of these 10 strategies by category (unlikely or amplified). Examination of these strategies reveals a common thread. Compared to those genuinely suffering from psychiatric disorders, malingerers present symptoms and other patterns of behavior that are deviant from what is typical, are rare, or exaggerated. In other words, compared with those actually suffering from disorders, malingerers seek to create in the minds of clinicians an impression of their affliction that often will overshoot the mark by not agreeing with actual symptom patterns of genuine cases.

**Table 1:** Detection strategies for malingered mental disorders.

**Category**	**Detection strategy**	**Strategy description**
Unlikely presentation	Rare symptoms	Focuses on endorsed symptoms that are reported very infrequently by genuine clinical patients. Malingerers often overreport these rare psychological problems
	Quasi-rare symptoms	Focuses on symptoms and characteristics that occur infrequently in normative (non-clinical) samples
	Improbable symptoms	Focuses on endorsed symptoms that are much more extreme than Rare Symptoms. This includes symptoms of a preposterous nature
	Symptom combinations	Focuses on symptoms and characteristics that commonly occur in genuine clinical patients, but that rarely occur in the combinations endorsed by malingerers
	Spurious patterns of psychopathology	An elaboration of Symptom Combinations. Utilizes particular scale configurations that detect patterns which are characteristic of malingering, but uncommon in genuine patients
Amplified presentation	Indiscriminant symptom endorsement	This strategy is based on the finding that some malingerers tend to endorse a large number of symptoms in comparison to genuine clients
	Symptom severity	In comparison to even severely impaired genuine patients, malingerers are more likely to endorse a large number of symptoms which they describe as being “unbearable” or “extreme”
	Obvious symptoms	This strategy focuses on the finding that in contrast to genuine patients, malingerers tend to endorse symptoms that clearly indicate a serious mental disorder
	Reported vs. observed symptoms	This strategy compares an individual's account of their symptoms to clinical observations. Malingering is often identified by clear discrepancies between endorsed symptoms and clinical observations
	Erroneous stereotypes	Focuses on common misconceptions that individuals have regarding symptoms commonly associated with mental disorders. Malingerers often overendorse these erroneous stereotypes

*Adapted from Rogers (19), and Rogers and Shuman (6)*.

Strategies for the detection of cognitive malingering differ from those used to detect the malingering of mental disorders, as they focus more on performance accuracy, although the detection of unusual response patterns applies to both domains (22–24). The two categories that classify detection strategies for cognitive malingering are *excessive impairment* and *unexpected patterns* (6, 20). Table 2 provides a description of each strategy by category. Responses detected by these strategies include performance failures on items that are typically achievable even by those with actual cognitive impairment and the detection of failure rates that are statistically unlikely. As before, a common thread across these different kinds of malingering, psychiatric or cognitive, is that malingerers seek to create false impressions in mental health professionals and often will miss the mark.

**Table 2:** Detection strategies for malingered cognitive impairment.

**Category**	**Detection strategy**	**Strategy description**
Excessive impairment	Floor effect	Uses very simple items that genuine patients are likely to successfully complete. Malingerers are likely to overestimate the difficulty of the items and consequently provide incorrect answers
	Forced-choice testing	Assesses for performances on cognitive tests that are lower than would be expected. Malingerers are identified by extremely poor performances
	Symptom Validity Testing	Utilizes a forced-choice format and assesses for below-chance performance and error rates that are statistically extremely unlikely
Unexpected patterns	Magnitude of error	Genuine patients often make predictable errors. This strategy identifies malingerers by focusing on high proportions of unexpected errors
	Performance curve	With continually increasing item difficulty, a predictable pattern emerges that is typically a negative curve when plotted on a graph. Malingerers are less likely to take item difficulty into account and therefore typically produce a different pattern
	Violation of learning principles	Based on established learning principles, this strategy identifies malingerers by comparing expected results to those that violate basic learning principles
Unexpected patterns (limited validation)	Consistency of comparable items	Compares performance with that of genuine patients by focusing on predictable patterns on comparable items within the same test. Malingering is likely to result in atypical patterns of performance. Requires rigorous testing across diverse samples, which is often unavailable for many assessments
	Psychological sequelae	Patients with genuine brain injuries often manifest additional symptoms. This strategy tests whether these additional symptoms differentiate malingerers from genuine patients. Caution should be used as malingerers may be able to recognize common sequelae
	Atypical presentation	This strategy assesses for unexpected findings (e.g., substantial performance variations on similar tests). Should be used with great caution as it lacks a firm conceptual basis

*Adapted from Rogers (19) and Rogers and Bender (20)*.

### Assessments of Psychiatric Malingering

#### Structures Interview of Reported Symptoms (SIRS)

SIRS is a comprehensive assessment for detecting feigned mental disorders, specifically an interview-based measure that consists of 172 items. A primary strength of the SIRS is its incorporation of multiple mental disorder detection strategies, including many of those identified in Tables 1, 2. Its primary scales include: Rare Symptoms, Symptom Combinations, Improbable and Absurd Symptoms, Blatant Symptoms, Subtle Symptoms, Selectivity of Symptoms, Severity of Symptoms, and Reported vs. Observed Symptoms [RO; (21)]. Five additional scales comprise the supplemental scales, producing a total of thirteen detection strategies, resulting in a particularly robust instrument. Items on the SIRS include Detailed Inquiries regarding symptomology and their levels of severity. Repeated Inquiries assess response consistency; and General Inquiries, which are designed to probe for specific symptoms, symptom patterns and general psychological disturbances (25).

The SIRS is the most commonly used and best-validated assessment in the forensic detection of malingering (11, 21). Although some research has suggested that the SIRS has low vulnerability to coaching, it is reported to produce lower specificity estimates than those reported in the official manual and has a higher rate of classifying true patients as malingerers than indicated by previous estimates [as cited in (26)]. Finally, many settings are inadequately equipped to utilize the SIRS given that the administration is complex, and the length of the interview can take significantly longer than the administration time of 30–40 min suggested by Rogers et al. (27–29).

#### Structured Inventory of Malingered Symptomology (SIMS)

The SIMS (30) is a paper-and-pencil screening devise for detecting malingering. Its items were drawn and revised from validity items of existent instruments and others were derived from research on attributes typical of malingerers. A 75-item scale, its subscales include psychosis, amnesic disorders, neurological impairment, affective disorders, and low intelligence. The SIMS yields a total score and subscale scores for each of the five subscales. Based on research with college students who were instructed either to malingerer or respond honestly, compared to other measures of malingering (e.g., the F and K scales of the MMPI), the SIMS total score has the highest sensitivity for detecting malingering (95.6%). Still, its validity in detecting malingering in more authentic contexts is largely unknown.

#### Minnesota Multiphasic Personality Inventory-2 (MMPI-2)

The MMPI-2 is a 567-item self-report measure designed to assess personality characteristics and psychopathology, although it is also used extensively outside of mental health and medical settings (31–35). The MMPI-2 has several validity scales designed to evaluate the accuracy with which test takers respond to test items and to predict distorted presentations. These include scales to detect the under- or over-reporting of symptoms.

Validity scales, developed to uncover malingering on the MMPI-2, include the F Scale (Infrequency), Fb scale (Back Infrequency), Fp Scale (Infrequency-Psychopathology), FBS (Symptom Validity), and Gough's Dissimulation Scale [Ds; (19, 36)]. Although the F Scale achieved the highest effect sizes among the various validity scales in two meta-analyses (37, 38), some researchers consider it to be inadequate considering it was designed only to detect atypical responding, which may also occur as a result of confusion regarding test items, a low reading level, or pathological interpretation of personal experiences (21). Many of the items on the F and Fb scales do not accurately distinguish between feigning and honest responding, with the Fb scale demonstrating poorer performance than the F scale (36, 39). Rogers and Neumann (36) concluded that both F and Fb are flawed scales for the detection of malingering. The most effective scales can misclassify 5–15% of individuals who attempt to malinger (40, 41). Heinze (42) reported even higher rates of false positives, stating that between 12 and 55% of individuals with genuine mental disorders have been identified by the MMPI-2 as malingerers.

#### Millon Clinical Multiaxial Inventory MCMI-III

The MCMI-III is 175 item self-report scale (true/false items) that takes about 30 min to complete (43). With a focus on personality disorders, its 28 subscales comprise the following categories: Modifying Indices (including validity items), Clinical Personality Patterns, Severe Personality Pathology, Severe Syndrome, and Clinical Syndrome. Atypical patterns, extreme scores, or high invalidity can suggest malingering (43).

#### Miller Forensic Assessment of Symptoms (M-FAST)

The M-FAST (44) is a brief screening measure designed to detect malingered mental illness in forensic settings by assessing individual response styles (45–47). The M-FAST contains 25 items, including 15 true or false questions, 5 Likert items, 2 yes/no questions, and 3 items designed to detect discrepancies between responses and observations (45, 47).

The M-FAST utilizes similar detection strategies as the SIRS, with four of its seven scales employing the same detection strategies (Reported vs. Observed, Extreme Symptomology, Rare Combinations, Unusual Hallucinations). It also contains three additional scales: Unusual Symptom Course, which assesses the reported speed of onset of mental illness; Negative Image, which capitalizes on the tendency of malingerers to believe that they should be viewed negatively by others; and Suggestibility, which relies on the likelihood that malingerers will endorse symptoms they believe will make them appear mentally ill (9, 48–50). However, a third of the scales on the M-FAST have low internal consistency, resulting in low reliability for these scales. Vitacco et al. (49) found problems with homogeneity for the individual M-FAST scales and lower utility estimates compared to the total score. In addition, they found that the M-FAST produced an unacceptably high rate of false positives (10%) using the total scale scores.

### Assessments of Cognitive Malingering

#### Tests of Memory Malingering (TOMM)

The TOMM is a recognition memory test that utilizes symptom validity testing (SVT), forced-choice, and floor-effect detection strategies. As a forced-choice SVT, the TOMM presents the respondent with two alternatives per test item, allowing for a 50% chance of choosing correctly. Scores falling significantly below this probability level suggest malingering (51, 52). As noted in Table 2, the floor-effect strategy involves the presentation of cognitive tasks which malingerers incorrectly believe impaired individuals are incapable of completing accurately (19). The TOMM contains 50 items and consists of two memory learning trials, with each trial followed by an assessment of recognition memory (53, 54). The respondent is initially shown a series of 50 line drawings, followed by a recognition assessment in which each drawing is presented alongside a foil. The subject is asked to identify the previously presented drawing and is given feedback regarding the correctness of the response (54–56). If the respondent does not achieve a correct score during the second trial on at least 45 items, a Retention Trial is administered. Malingering should be suspected if the respondent earns a score of 45 or less on the second trial or the Retention Trial (53, 56). Some researchers have reported lower hit rates with the TOMM than with other measures. Unfortunately, high face validity enables a large number of respondents to perceive it correctly as an assessment of malingering (57).

#### Rey Fifteen-Item Test (FIT)

The FIT utilizes the floor effect detection strategy but without a forced-choice design (6, 19, 58, 59). The FIT presents a memory task that appears difficult but is actually easy. The individual is shown 15 different items consisting of letters, numbers, and geometric shapes for a brief period and then asked to recall and reproduce as many of the items as possible (58–61). The fifteen items are presented in five rows containing three items each. The first row presents the numbers 1, 2, and 3; the second presents the roman numerals I, II, and III; the third presents a square, a triangle, and a circle; the fourth presents the letters A, B, and C (Capitalized); and the fifth presents the letters a, b, and c, all in lowercase (61). A cut-off score of nine is most commonly used (54), although some have suggested the use of lower cut-off scores of eight or less to accommodate those with true impairment (62). Schretlen et al. (63) concluded that the FIT has several limitations and that patients with genuine impairment often perform poorly on the test, while many malingerers score above the recommended cut-off score. A number of studies have shown that forced choice recognition tests are more useful in identifying cognitive malingering than the standard FIT. Clinicians should also note that the FIT does not meet the Daubert standard, which outlines criteria for the admissibility of scientific evidence in court (64–66).

#### Word Memory Test (WMT)

The WMT is a forced-choice test of malingering. In addition to the forced-choice detection strategy, it also utilizes the following: (a) violation of learning principles, (b) floor effect, (c) symptom validity testing, and (d) the performance curve (6, 19, 57), all noted in Table 2. The learning principle it utilizes is the advantage of recognition memory performance over recall, which malingerers may not account for in their efforts to deceive. The WMT is more effective than other measures of feigning in its use of this detection strategy, yielding large effect sizes (19).

Regarding administration, the respondent is presented with 20 pairs of semantically-related words during two learning trials. Immediately following these presentations, the Immediate Recognition trial begins in which each of the 40 words is paired with a foil and the individual is asked to select the correct target word. After 30 min, the delayed recognition trial is given, and target words are paired with new foils. Four separate effort tests, designed to evaluate verbal memory, are then given, including the multiple choice, paired associates, delayed free recall, and long delayed free recall subtests. Scoring is accomplished by comparing the number of words recognized consistently across the immediate and delayed trials. A score of 82.5% or below is the cut-off (54, 55, 67). Although simulated malingerers perform worse than participants instructed to perform at their best on the WMT, coaching and the use of sophisticated simulators has resulted in less accurate detection of malingering with this instrument (67, 68). Pella et al. (59) warn that the WMT may be particularly vulnerable to coaching compared to other instruments, resulting in a high rate of false negatives.

## Lie Detection

Despite advances in malingering detection technology, current methods are far from adequate, with high rates of false positives, false negatives, and a susceptibility to coaching. Perhaps the detection of malingering can be facilitated by incorporating developments from the field of lie detection given that malingering is high-stakes deception. Current methods of lie detection are reviewed, with an emphasis on innovative cognitive-based approaches.

### Human Lie Detectors

Although lying is common in everyday life (69, 70), people are amazingly poor lie detectors. Individuals accurately judge lying at or slightly above chance levels but are a bit better at identifying truth telling (71–75). Although one might assume that professional lie-catchers (e.g., police officers, customs officers, judges, mental health professionals) have better accuracies at detecting lies, the majority of studies show that they do not (73, 76–79). Rather than having to depend on unreliable human lie detectors, we now review some prominent and emerging technologies potentially applicable to ferreting out malingering, many with minimal dependence on human lie detectors.

### Arousal-Based Approaches

#### Control Question Technique

The *polygraph* is a scientific instrument that continuously records psycho-physiological arousal as assessed by pulse rate, blood pressure, respiration rate, and/or skin conductivity, which has been applied to the detection of deception. The most common questioning procedure used with it is the Control Question Technique (CQT; 79). In a typical test, a respondent is given a pretest interview for gathering information that provides the basis for control questions. Once questions are constructed, the examiner will preview them with the respondent to ensure that they are understood and will not surprise the respondent when asked later. During the examination, irrelevant questions are asked such as “What is your age?,” along with the control questions that most people tend to lie to. For example, “Have you ever stolen anything from your place of employment?” Finally, relevant questions, probing the issue central to the exam, are asked (e.g., “Did you rape … on January 7th?”). The questions usually elicit brief answers. A guilty liar, it is hypothesized, will show more arousal to relevant questions than to control questions, whereas an innocent, honest respondent will show more arousal to control questions (80). Law enforcement and federal agencies in the United States use the CQT as a screening device for hiring and retaining employees and as a tool for criminal investigations. The CQT has been used to verify victim's statements, evaluate the veracity of witnesses, and to exonerate suspects. Still, test results are largely inadmissible in US courtrooms (81).

A major criticism of polygraph-based techniques, especially the CQT, regards their generally poor validity. Specifically, the CQT produces a high rate of false positives, that is, the labeling of honest individuals as liars (81–84). Researchers have also found that respondents can easily be trained to evade detection by using mental and physical distraction techniques known as countermeasures (81, 84).

#### Concealed Information Test (CIT)/Guilty Knowledge Test (GKT)/Concealed Knowledge Test (CKT)

Partly in response to the validity concerns with the CQT, the CIT, also known as the CKT and GKT, was proposed. It is a questioning paradigm that can be used with the polygraph to uncover the false denials of respondents by exposing whether they possess guilty knowledge or concealed information, presumably resulting from their participation in a crime or some other experience (80). During a typical CIT, the respondent is presented with multiple-choice questions, each having one relevant alternative (correct answer) and several neutral alternatives (plausible distractors). The latter should be chosen such that an innocent person could not discriminate them from the relevant alternative (80). An example of a relevant question is “How was the victim killed?,” with the response alternatives of “shot,” “stabbed,” “struck,” “strangled,” or “poisoned.” This question could be re-asked multiple times, along with other questions probing different aspects of a crime scene. The respondent need not answer. If heightened arousal occurs consistently to relevant responses, then the respondent may be concealing information as the perpetrator. The CIT assumes that innocent respondents could not have acquired guilty knowledge indirectly and that guilty respondents encoded guilty knowledge and have retained it (85).

Some validity concerns with the CQT were resolved in the CIT, including more standardization of the procedure, more appropriate control alternatives, fewer false positives, and a stronger theoretical basis (80). Also, beyond the psycho-physiological measures of the polygraph, concealed information has been uncovered with the diverse cues of response time (86–90), event-related potentials (91–93), and pupil dilation (94). Also, the CIT has been used to expose the simulation of amnesia (95). Still, the CIT is limited in the deception it can uncover to the false denials of those possessing concealed knowledge.

### Cognitive Load-Inducing Approaches

Cognitive load refers to the demands made on the limited pools of attention and working memory resources for performing mental tasks (96, 97). Some recent, novel, and promising techniques for detecting deception, and possibly malingering, rather than viewing deception as a physiological/emotional event as does the CQT, view it as a cognitive act that generally imposes greater cognitive load on respondents than honesty does. In support, Vrij and Mann (98) reported that telling complex, high-stakes lies increased cognitive loads, with liars exerting significantly more effort to control their speech than did truth tellers. As further neurological support, brain imaging studies using fMRI (functional magnetic resonance imaging) scanners, which reveal brain activity during task performance, suggest that deception activates higher brain centers associated with cognitive demand, particularly in the frontal lobe (99, 100). If lying is more cognitively demanding than truth telling, deception should reveal itself in longer times needed to answer questions, more inconsistencies and hesitancy in answering logically interrelated questions, greater pupil dilation, more activity in the brain's prefrontal cortex, more blinking, and in other signs of heightened cognitive load.

Cognitive load-inducing lie detection techniques, only some of which can be reviewed due to their sheer number, seek to enhance the mental effort of liars compared to truth tellers, in effect, making it mentally harder to deceive than to be honest (101, 102). Once refined and validated, such techniques may accurately expose malingering in forensic settings, perhaps used in conjunction with existing methods.

#### Asking Surprise Questions/Soliciting Surprise Drawings

Asking surprise questions of respondents can increase cognitive load on liars. For instance, Vrij et al. (103) instructed pairs of participants to lie or tell the truth about whether they had lunch together. All pairs then prepared for an interview that followed, which included anticipating likely questions. During the interview, general and unanticipated questions were asked, some of the latter probing minor details like these: “What was the color of the shirt your partner wore?” “Who sat closest to the door?” Inconsistencies across such questions from members of each pair allowed observers to classify liars and truth tellers beyond chance, as did discrepancies across surprise pictures that members were asked to draw of the layout of the restaurant. Although researchers did not measure the cognitive loads produced by the surprise questions or drawings directly, we regard them as cognitive load-inducing because deceptive participants likely had to think more than truth tellers to guess at how their partners might respond to the questions to ensure their answers and drawings would be consistent (104).

These results are promising. Still, asking surprise, detailed-oriented questions has limitations. Once knowledge of this lie detection technique disseminates, liars may include spatial and other obscure details into their deceptive narratives in anticipation of surprise questions. Also, memory for minor details can go unnoticed by truth tellers (105). Thus, if respondents claim “I can't remember” to detail-oriented questions, they may be answering honestly. Similar concerns apply to drawing pictures. Even so, refinement of these techniques may overcome these concerns.

#### Having to Maintain Eye Contact

Having to maintain eye contact with another can selectively heighten cognitive load and anxiety in liars. In support, Vrij et al. (106) directed some participants to lie to interview questions while others told the truth. Some of the participants were also directed to maintain continuous eye contact with the interviewer. Interviews were videotaped and observers of the recordings were more accurate at discriminating liars from truth tellers when eye contact had to be maintained, suggesting that doing so induces higher load and anxiety in liars than in truth tellers, perhaps because eye contact is distracting to liars who need to focus their attention inwardly to construct plausible deceptions.

One likely countermeasure, as knowledge of this load-inducing technique spreads, would be to practice lying while maintaining eye contact with another, which might reduce liar-truth teller differences. Even so, combined with other techniques, it may be useful in revealing malingering in forensic contexts.

Rather than heightening cognitive load through surprise or by imposing a concurrent task (e.g., maintaining eye contact), the two techniques described next (aIAT, TARA) add to cognitive load by creating response interference in deceivers by having them respond quickly and accurately to some items intermixed with others they may want to lie to. Such techniques also allow automated lie detection, not dependent on unreliable human observers.

#### Autobiographical Implicit Association Test (aIAT)

Based on the Implicit Association Test of Greenwald et al. (107), the aIAT is designed to determine whether respondents possess actual autobiographical memories, for instance, of a true alibi at the time of a crime. This computerized, forced choice assessment confronts respondents with five blocks of sentences to be classified (108). In block 1, respondents classify sentences with verifiable truths as true or false. In block 2, target sentences probing specific episodic truths (guilty if true; innocence if false) about them are likewise classified. Blocks 3 and 5 are crucial. In block 3, true and guilty sentences are intermixed and classified with the same response key. In block 5, true and innocent sentences are likewise classified together. An index, D, which penalizes for incorrect responses, is largely based on subtracting block 3 response times from those of block 5. Positive D scores are expected of guilty respondents, negative D score of innocent because of the interference in guilty respondents caused by the incongruence of combining truthful and innocent responses in block 5.

The aIAT has an impressive 91% accuracy rate in identifying those possessing genuine autobiographical memories (108) and has proven effective in uncovering the malingering of whiplash (109), and unveiling phantom limb pain (110). Clearly those genuinely affected by cognitive or psychiatric impairments should have many life memories of experienced symptomology that can be probed. Still, the aIAT has some limitations. It does not allow for ascertaining the truths of answers to specific or open-ended questions (e.g., When did you first notice your memory problems?). Also, research has not adequately explored whether countermeasures, such as deliberately slowing on some blocks and speeding up on others, could reduce deception detection (108). Another limitation, the aIAT requires the possession of true identity information that can be contrasted with faked identity information. In the case of those seeking to fake their identities in the field, such information may be unavailable to examiners (111).

#### The Timed Antagonistic Response Alethiometer (TARA)

Like the IAT and aIAT, TARA (112) involves a multi-block classification task. This computer-administered, response time-based method of lie detection assumes that, following instructions to minimize errors, incompatible tasks take longer to execute than compatible ones. Statements are presented on a computer screen that respondents must quickly classify as true or false. At first, control statements with verifiable truths (e.g., Rocks are hard. Mozart wrote novels.) are presented. In blocks that follow, target statements probing truths specific to the individual (I am male. I am a citizen of Egypt.) are presented. When target and control statements are combined within the same block, dishonest respondents experience response interference and the longest response times, having to perform the incompatible responses of deception and truthfulness. TARA correctly classified liars and truth tellers with an accuracy of 85%.

TARA differs from the aIAT in some important ways. TARA uses two categorizations (true, false) rather than four, uses only one critical block rather than two, and identifies lying from truths based on absolute RTs in the critical block. The latter requires comparison with a matched control group, a limitation of this technique (108). Still, TARA has potential to uncover a variety of deception types, including malingering. However, like the aIAT, it does not support the verification of open-ended responses or an answer to a particular question, nor has it been applied to detect deception involving a specific issue such as participation in a crime. Also, the effects on detection accuracy of the extensive practice of deception, deliberate slowing on certain blocks, or the use of other countermeasures are unknown.

#### Detecting Faked Identities With Unexpected Questions and Mouse Movements

The aIAT and TARA use key press response times to uncover deception. In order to discover faked identities in a way not reliant on possessing accurate identity information about respondents, Monaro et al. (111) explored the use of computer mouse movements in responding yes/no as the cues to deception in conjunction with asking unexpected questions. Measuring mouse movements allows a much richer set of behavioral cues, such as acceleration and trajectory, not easily controlled via countermeasures. Investigators assigned participants either to rehearse their true identities or rehearse then lie based on fake identities. Expected questions (i.e., concerning rehearsed information, such as birth month) and unexpected questions (e.g., one's Zodiac sign), were asked, the latter hypothesized to be constructed impromptu under high cognitive load. Detecting an impressive 95% accurately, fakers took longer, especially in responding to unexpected questions, and had longer response trajectories, among other differences.

Asking unexpected question, [see (113)] combined with mouse movements, has much potential, for instance, in detecting faked depression (114). However, would it be effective in uncovering malingerers who have faked depression or other psychiatric disorders for years? Also, the guidelines for generating unexpected questions are unclear. For example, a truth teller, not inclined toward superstition, might lack quick access in memory to their Zodiac sign. Verifying answers to open-ended questions is not possible as well. Even so, it is interesting to consider the kinds of unexpected questions that might blindside malingerers (e.g., Does your impairment affect you when driving?) and expose them.

#### Time-Restricted Integrity Confirmation (TRI-Con)

Walczyk et al. (115) proposed a cognitive load-inducing technique, *Time Restricted Integrity-Confirmation* (TRI-Con), with potential to uncover different kinds of deception including malingering. It is based on a cognitive theory of high-stakes deception called Activation-Decision-Construction-Action Theory (ADCAT), summarized later. Like the aIAT and TARA, TRI-Con can be largely automated via computer-administration and scoring and selectively enhances the cognitive load on liars by adhering to seven guidelines during lie detection examinations (115, 116).

The guidelines are: (a) Respondents are prompted about the focus of the question set to follow (e.g., “The next 15 questions concern your activities and whereabouts at the time of the crime.”). By priming relevant episodic and semantic truths, prompts reduce respondents' need to search memory to tell the truth, making cognitive load indices clearer cues of when respondents are constructing lies. As with reviewing questions before a polygraph exam, prompting also reduces the emotional surprise accompanying blindsiding respondents with questions that probe sensitive issues. (b) Still, the specific questions are not disclosed until asked during an exam, thus surprising respondents cognitively and reducing the chance that deceptive answers were prepared and rehearsed. (c) Questions, both yes/no and open-ended, are written when possible to be unclear regarding what truths are sought until fully asked, which should reduce respondents' chance of preparing deceptive answers as questions are being asked. (d) To obtain clearer assessment of the cognitive load needed to answer completely, questions are written to be answerable, as much as possible, with one or a few words. (e) Respondents are instructed to answer as quickly as possible to discourage and expose attempts to deceive. The high cognitive load of rapid responding to surprise questions may also increase cue leakage in the form of voice pitch elevation, pupil dilation, increased blinking, and long response times because of the limited opportunity for liars to monitor and control their own behavior (75, 117, 118) and may increase accidental blurting of the truth (119). (f) Without adequate preparation, liars' deceptive accounts should be incomplete. Questions are asked and then re-asked, along with logically interrelated questions, to increase liars' cognitive load and provoke inconsistencies (120). (g) Behavioral baselines for ground-truth answers are established for all cognitive load indices for comparison with levels of these cues of answers suspected of deception. This practice controls for individual differences in behavioral base rates and improves the accuracy of lie detection (71).

Given the inaccuracy of human lie detectors (71, 72), automatable techniques of lie detection, such as TRI-Con, TARA, and the aIAT, provide auspicious alternatives. For instance, with TRI-Con questions can be recorded and asked by a computer. Using microphone-headsets, answer response times can be precisely measured to the millisecond level of precision. Connected modern eye-tracking systems can concurrently measure pupil dilation, eye movements/fixations, and blinking rate. Voice pitch elevation can be detected using the appropriate software, etc.

Following the guidelines above, studies have shown the effectiveness of TRI-Con for uncovering deceptive answers to yes/no and open-ended questions. Walczyk et al. (115) instructed adults to lie or tell the truth to questions about various aspects of their lives such as employment history and their performance on standardized tests. Using response time as the cue, discriminant analyses allowed classification of liars and truth tellers above chance. Likewise, Walczyk et al. (116) tested TRI-Con again by asking participants to lie or tell the truth about their lives and included a rehearsal condition in which participants prepared deceptive answers, a likely load-reducing countermeasure. The consistency of answers across interrelated questions was added as a cue. Liars and truth tellers were classified up to 89% accurately. Analyses also showed that the countermeasure of rehearsing deception is detectable. Also, Walczyk et al. (121) tested TRI-Con in a forensically-relevant context. “Witnesses” observed actual crime videos, then later told the truth or lied, rehearsed or unrehearsed, when interviewed about them. The cognitive cues were response time, answer consistency, eye movements, and pupil dilation. Discriminant analyses allowed classification of the three conditions 69% accurately, 33% expected by chance. Truth tellers generally had moderate response times, the fewest inconsistencies, and the most eye movements. Regarding the latter findings, liars appeared to move their eyes less to avoid visual distraction that would have heighten cognitive loads as they focused attention inwardly to construct lies. Walczyk et al. (122) observed similar results for participants who lied or told the truth concerning their participation in a mock crime. Across these studies, low rates of false positives were observed, recalling that high rates are a perennial problem with the CQT (81).

Although TRI-Con has potential for the detection of malingering, it too is susceptible to the countermeasure of rehearsal. The good news is that load-reducing techniques can be combined. TRI-Con already involves surprise questions. Respondents can be further instructed to maintain eye contact with someone present. Surprise drawing can be added after the exam to solicit non-verbal information. Other load-inducing techniques can be added. Combining several load-inducing techniques within lie detection exams and assessing several indices of cognitive load should make the detection of malingering hard to foil.

## Activation-Decision-Construction-Action Theory (ADCAT)

A major criticism of the polygraph-based CQT is its lack of a valid theoretical foundation (80, 81). Similarly, most existent load-inducing techniques assume that lying is more cognitively demanding than truth telling. Our discussion of the countermeasure of rehearsing deception, however, suggests that this is not always true. No coherent theory underlies most of these techniques. TRI-Con is an exception, based on ADCAT, a theory of high-stakes deception. ADCAT, with some tweaking, might account for malingering. Such a theory, once validated, could suggest cues of when malingering has taken place and new ways of detecting it. The most recent version of ADCAT, Walczyk et al. (123), is summarized below, with an emphasis on its application to malingering.

ADCAT accounts for how individuals respond deceptively to solicitations of the truth, such as a question, under high stakes. A high stakes social context is one in which being honest with targets (those soliciting truths) would likely prove very costly to respondents in the non-attainment of goals important to them. High-stakes contexts include a perpetrator interrogated by a detective concerning an alibi or a psychiatrist assessing a sane perpetrator regarding his fitness to stand trial.

ADCAT specifies four psychological components involved in most instances of deception. Each elaborates on underlying cognitive processes. ADCAT incorporates established concepts of cognitive science, including *working memory* and *executive functioning* (123). Of central importance is *Theory of Mind* (ToM), which involves the inferences individuals make regarding the mental states of targets. First-order ToM inferences in deception entail the false beliefs that liars are trying to create in others (e.g., “I want this psychiatrist to believe that I cannot distinguish right from wrong.”). More abstract and cognitively demanding second-order ToM inferences concern, for instance, malingerers' guesses of what targets will expect in them if their deceit is believed (“How should I behave and what should I say to come across as legally insane?,” (124, 125). As noted, malingerers are often wrong in these guesses. Both types of inferences are heavily involved in all four components.

### Activation Component

The first component of ADCAT, *Activation*, involves the retrieval of the truth following targets' solicitations of accurate information. For instance, a police detective might ask a perpetrator who is feigning memory loss whether she can remember even a small fragment regarding where she was when the crime occurred (123). Based on the social context and roles targets play, ToM inferences are made regarding why, for instance, the detective is seeking the information, to what use sharing the truth will be put, etc. (e.g., “This detective suspects me and wants to build a case to charge me.”). Most truths are automatically activated by a question but occasionally must be searched for in LTM if they have not been accessed in a long time or may need to be newly constructed in WM, both of which can add to the cognitive load of truth telling.

### Decision Component

Typically with the truth now active in WM (126, 127), the second component, *Decision*, will execute. It describes how respondents choose whether and then how to deceive. With the help of ToM inferences, respondents will first evaluate what the likely overall gain/loss is of sharing the truth vis-à-vis the non-attainment of important goals such as staying out of prison or maintaining their disability income. Such evaluations are made intuitively when deception is impromptu but can be more deliberate when high-stakes truth solicitations are anticipated. These calculations involve intuitively combining estimates of the likelihoods of salient outcomes with their *subjective utilities*, that is, the personal value of the outcomes to respondents (128). The more negative the expected overall loss, the more likely a deception will be considered (123). In such a case, one or more context-appropriate deceptions will be evaluated in terms of their overall likely gain/cost vis-à-vis their believability and how well each helps respondents to achieve their goals. Again, first- and second-order inferences are crucial to accurately evaluate the likely impact of deceptions on targets.

The deception with the highest expect gain, if any, will be chosen, which can vary from sharing a truth with an important detail withheld (lie of omission) to a bald-faced lie (complete fabrication). The preference for respondents will be to minimize the deception needed to attain their goals (129). The decision to deceive intrinsically adds to cognitive load (115), an implication of which is that surprising respondents with questions will require them to decide impromptu whether to lie, enhancing the mental work of deception and related cognitive cues.

### Construction Component

During the third stage, *Construction*, the specific deception chosen is elaborated as needed to go undetected and achieve respondents' other goals. The cognitive load imposed varies with the type of deception. A false denial or a lie of omission can impose minimal load whereas constructing a bald-faced lie, for instance, a false alibi for what happened at the time of the crime, can impose the greatest. Especially for the latter, second-order ToM inferences must be made to ensure that a lie is internally consistent, consistent with what targets' know or are likely to find out, and detailed enough to be believable (123). A chance to prepare deceptions in advance of delivery will make them more believable, internally consistent, etc., and allow respondents to anticipate likely questions from targets (130, 131). A relevant question for the detection of malingering during this component concerns what kinds of ToM inferences do malingers typically make to mislead mental health professionals in forensic contexts. Little research has addressed this question. Asking surprise and complex questions of respondents suspected of malingering under TRI-Con concerning lesser known actual symptoms of disorders might trip up malingerers, producing long response times and other signs of cognitive load compared to those actually afflicted.

Central to the construction component is the *plausibility principle*, which specifies the order of steps respondents generally will take to construct believable deceptions, especially the bald-faced variety. Respondents will (a) first attempt to modify the truth, related episodic memories, or other personally experienced memories based on second-order ToM inferences of what targets will believe (102, 123, 129, 131). Because recently accessed memories are more retrievable, they will be preferred to distant memories (132). If respondents have no such memories, for instance, because malingerers have never actually suffered from a particular mental disorder, they may (b) use schemata or scripts of what is typical within that context to provide the basis of the deception (132–134). If such schemata are unavailable, again due to limited life experience or if relevant schemata are inaccessible, respondents will (c) construct deceptions using assorted information accessible from LTM as cued by the social context, which imposes the highest cognitive load. To summarize, the plausibility principle predicts that cognitive load will increase when going from *a* to *c* as the basis of a deception and lie plausibility will tend to decrease. However, the opportunity to prepare and rehearse deceptions, for example, a false presentation of being insane, in advance of delivery is a countermeasure that can lower the cognitive load experienced by liars, even below that of truth tellers (116, 135). On a positive note, the use of such rehearsal may be detectable by cognitive load indices falling below levels of truth telling (116).

### Action Component

During the final component, *Action*, respondents deliver the lies they have prepared, or will generate impromptu, to targets. In general, they will attempt to control physical movements and appear relaxed but may self-regulate too much because of inaccurate ToM beliefs they hold about the actual behavior of truth tellers. Many liars naively implicitly assume that honest individuals are relaxed and do not experience recall failures or make other mistakes in conveying truths (72, 104). As noted, the cognitive load of delivery will decrease for well-rehearsed lies, but will increase when social contexts are unfamiliar and complex. Cognitive load will also increase when, for instance, malingerers are surprised by truth solicitations, which allows little time for lie preparation (115).

Because deception is typically chosen only when honesty blocks goal attainment, truth telling is usually more practiced and automatic (129). Especially for well-rehearsed truths, conveying corresponding deceptions can impose a cognitive load during delivery, requiring active suppression of the truth (100, 116, 136, 137). In addition to this source of cognitive load, lies told in high-stakes situations are highly motivated, which can heighten the fear of being caught as well as the cognitive load of delivery (138). ADCAT hypothesizes that impromptu liars will manage the increased load of deception by minimizing eye contact (106), reducing eye movements (122), reducing body movements, occasionally scanning the environment for lie construction hints, and implementing time-buying strategies like asking for a question to be repeated or pausing before and during delivery of the lie (123).

## Applying Cognitive Load-Inducing Technique and ADCAT to Detect Malingering

Only sketched above, ADCAT advances understanding of the behavioral manifestations of deception by providing a detailed cognitive account of the processes individuals engage in as they choose deception, construct lies, and deliver them to targets (123). Professionals interested in advancing the cognitive detection of malingering are encouraged to learn more about this and other cognitive accounts of deception [see (84, 139)]. Malingering is high-stakes deception in which malingerers must actively inhibit the truth (e.g., a lack of mental illness) and decide which deceptive presentation of behavior to construct and practice. Interestingly, constructing presentations of feigned mental disorders may be more cognitively complex than constructing, for instance, alibis based on complete fabrications. Second-order ToM inferences are likely extensively made as malingerers study the kinds of symptoms typical of those afflicted with particular disorders and how the disorders are assessed. ADCAT helps clarify when cognitive load-inducing approaches for detecting malingering are likely to be effective. For instance, ADCAT recognizes the preparation and rehearsal of high-stakes deception before delivery as the most likely foil of such approaches and recommends that respondents be blindsided with memory tasks for accessing truths. These include asking unanticipated and complex questions, soliciting surprise drawings, or accessing memories for events in unusual ways like recounting an alibi in reverse-chronological order (140). In such cases, the cognitive load of deception should exceed that of honesty. Surprisingly, most researchers who have tested load-inducing approaches have not given much attention to the countermeasure of rehearsal.

The customary methods for malingering detection we reviewed rely on atypical levels or combinations of symptoms, unusual performance on cognitive tasks, or other behavioral anomalies. Sadly, their rates of false positives and false negatives tend to be high. As an alternative, we encourage those interested in detecting malingering in forensic contexts to consider combining several cognitive load-inducing approaches like those we discussed. For instance, TRI-Con automates many aspects of lie detection, involves surprise questions, and can include maintaining eye contact and other load-inducing techniques. The non-load-inducing cognitive methods of lie detection of Criteria Based Content Analysis (CBCA) and Reality Monitoring (RM) can be added as well, which assume that liars fabricate information when constructing lies (73, 141). Both attempt to differentiate memories of real experiences from fabrications by assessing for features of authentic experiences such as sensory details, the reporting of unexpected complications, thoughts or feelings experienced, contextual information, temporal details, and affective information (98, 141). Under TRI-Con, asking respondents surprise and complex questions about details like these related to their disorders, their time of onset, or how they made respondents feel might expose significantly higher cognitive loads in malingerers than in genuine patients as longer response times, elevated pitch, dilated pupils, less eye movement, or as slower and longer mouse movements (111). In conclusion, the more that malingering is understood cognitively, the more that innovative methods of lie deception detection like TARA, the aIAT, and TRI-Con can be refined to supplement existing assessments.

## Author Contributions

All authors listed have made a substantial, direct and intellectual contribution to the work, and approved it for publication.

### Conflict of Interest Statement

The authors declare that the research was conducted in the absence of any commercial or financial relationships that could be construed as a potential conflict of interest.
